# Candida Spondylodiscitis After Transforaminal Epidural Steroid Injection: A Case Report

**DOI:** 10.7759/cureus.95392

**Published:** 2025-10-25

**Authors:** Hyeryung Kang, Jeongeun Lee

**Affiliations:** 1 Department of Anesthesiology and Pain Medicine, VHS (Veterans Health Service) Medical Center, Seoul, KOR

**Keywords:** candida albicans, discitis, epidural injections, invasive fungal infections, spondylitis

## Abstract

Transforaminal epidural steroid injection (TFESI) is a widely used intervention for lumbar radicular pain. Although rare, fungal spinal infections can follow such procedures and require prompt diagnosis and management. A 76‑year‑old male developed progressive back pain and bilateral leg weakness two weeks after bilateral L5 TFESI. He received empirical antibiotic therapy in an outside emergency department, but showed no progress. He subsequently presented to our outpatient clinic 11 weeks after TFESI. MRI showed pyogenic spondylitis and osteomyelitis at L1-L2 with psoas involvement. No pathogen was identified in diagnostic evaluations, including acid-fast bacillus (AFB) staining of blood and sputum, blood cultures, urine culture, microorganism identification tests, and bone biopsy cultures. Worsening motor weakness prompted L1-2 decompression; intraoperative disc cultures grew *Candida albicans*. After discontinuation of empirical antibiotics and initiation of intravenous fluconazole, his neurological status improved; beginning the day after surgery, his lower limb motor strength increased from grade 2 to grade 4, and he was discharged to a rehabilitation facility with motor strength of 4. This case underscores the need for early microbiological diagnosis and surgical biopsy in post‑procedural spinal infections unresponsive to empirical antibiotics. Clinicians should consider fungal etiologies, especially in elderly or oncologic patients. To the best of our knowledge, this represents one of the first documented cases of *C. albicans* spondylodiscitis following TFESI in an immunocompetent patient.

## Introduction

Transforaminal epidural steroid injection (TFESI) is a widely used interventional procedure for lumbar radicular pain. While generally considered safe, infectious complications such as spondylodiscitis and vertebral osteomyelitis can rarely occur [1]. Most reported cases involve bacterial pathogens such as *Staphylococcus aureus*; however, fungal infections of the spine are increasingly being reported, particularly among immunocompromised individuals or those undergoing invasive spinal procedures [2,3]. *Candida albicans* is an unusual but important cause of spondylodiscitis, and delayed identification can result in treatment failure and neurological decline [4,5]. This case highlights the challenges of diagnosing fungal spinal infections in a non-immunocompromised patient and the critical role of early surgical intervention in both diagnosis and management.

## Case presentation

A 76-year-old male patient underwent bilateral L5 TFESI and developed severe back pain with bilateral leg weakness two weeks later. One week after symptom onset, he began visiting an outside emergency department daily, where he received empirical intravenous antibiotics. It was initiated in the absence of an identified pathogen. Based on the empirical treatment principles outlined in the 2015 IDSA Clinical Practice Guidelines for Native Vertebral Osteomyelitis [6], initial therapy was started with agents active against common pyogenic pathogens. In this context, cefditoren was administered for two weeks, followed by ciprofloxacin with clindamycin for an additional two weeks, and subsequently teicoplanin with ceftriaxone for another two weeks. However, these sequential modifications, made in response to the lack of clinical improvement and progressive worsening of symptoms, failed to halt his deterioration. He subsequently presented to our outpatient pain clinic 11 weeks after the initial TFESI.

**Figure 1:**
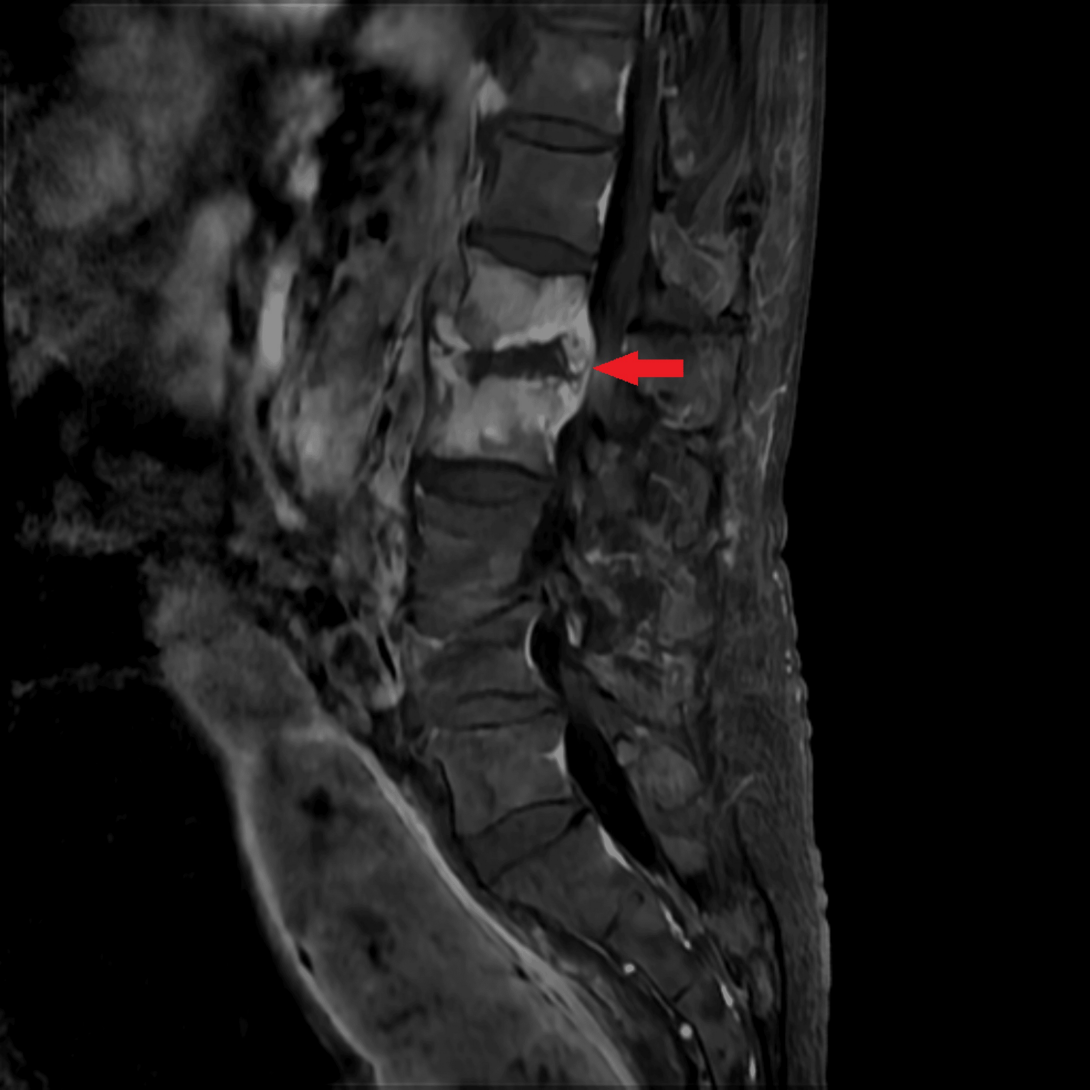
Initial enhanced magnetic resonance imaging. The L1–2 vertebral bodies show increased signal intensity, suggestive of osteomyelitis and discitis.

His past medical history was notable for non-small cell lung cancer, atrial fibrillation managed with rivaroxaban, and severe mitral regurgitation. He was admitted immediately for further evaluation and management. At that time, he was non-ambulatory with mild bilateral lower limb edema. Enhanced lumbar magnetic resonance imaging (MRI) demonstrated spondylodiscitis and osteomyelitis at L1-L2 with bilateral psoas extension (Figure 1), and his C-reactive protein (CRP) level was elevated at 38.68 mg/L.

Neurological examination revealed motor strength of grade 3/5 in both lower limbs. Blood cultures were drawn following MRI to evaluate for possible bacteremia or systemic infection, and whole-body cultures, including sputum, urine, and stool, were done but came out negative. To facilitate pathogen identification, antibiotics were discontinued for three days, and an L2 vertebral body bone biopsy was performed one week after admission. Comprehensive tests were conducted, including tissue Gram stain and culture, tissue AFB stain and culture, *Mycobacterium tuberculosis* polymerase chain reaction (MTB PCR), periodic acid-Schiff stain (PAS stain), and fungal culture using standard bacterial enrichment broth media; no organism was isolated. Subsequently, his neurological status worsened, accompanied by an increase in CRP to 151.34 mg/L with an erythrocyte sedimentation rate (ESR) of 77 mm/hour and white blood cell (WBC) count of 9.7 × 10³/µL and a decline in motor strength to grade 2/5. 

Five days after the biopsy, unilateral laminotomy for bilateral decompression (ULBD) and discectomy were performed at L1-2 via a left-sided approach. Intraoperatively, a grayish, inflamed extruded disc was observed, causing compression of the thecal sac and the left L2 nerve root at the T12-L1/L1-2 level. A laminectomy at the left T12 and L1 was carried out for adequate decompression.

**Figure 2:**
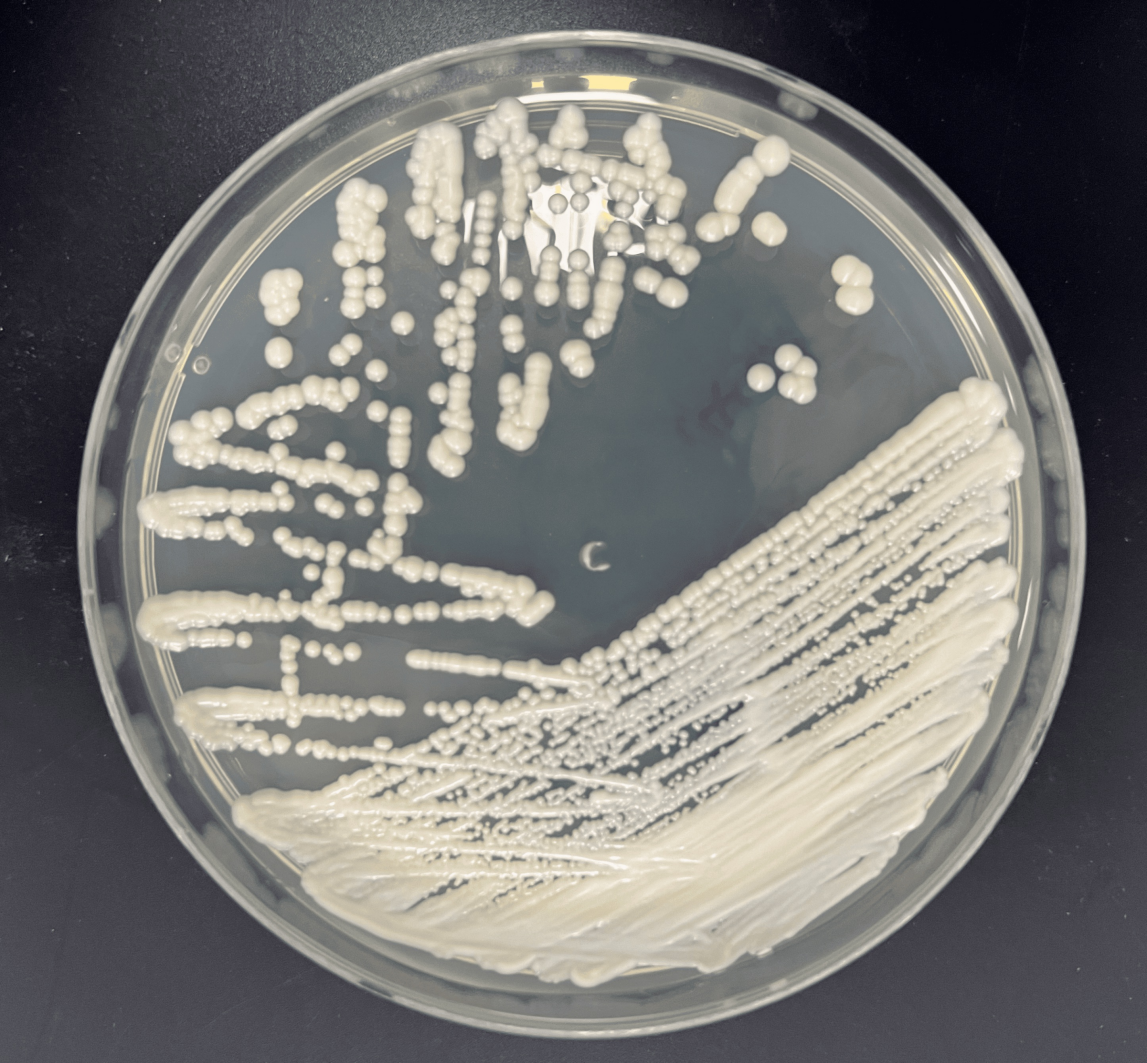
Candida albicans grown on a blood agar plate. Creamy, smooth yeast-like colonies are visible, characteristic of fungal growth.

Following neural decompression, posterior fixation was performed with four pedicle screws inserted bilaterally at L1-2. Intraoperative cultures of disc material grew *C. albicans* (Figure 2). All antibiotics were discontinued, and intravenous fluconazole therapy was initiated. The patient demonstrated rapid improvement following antifungal treatment, with ESR decreasing to 69 mm/hour, CRP to 44.13 mg/L, and WBC count to 8.57 × 10³/µL after three days. Beginning the day after surgery, his lower limb motor strength improved from grade 2 to grade 4, and he was discharged to a rehabilitation facility. At the three-month follow-up, he maintained grade 5 strength and was able to walk independently, with no recurrence of infection on MRI.

## Discussion

Infectious spondylitis following transforaminal epidural injection is rare but can have serious consequences if not promptly and accurately diagnosed. The most common pathogens are *S. aureus* and *Escherichia coli*, while fungal organisms are infrequent but increasingly recognized. Nevertheless, they have been responsible for occasional large outbreaks, most notably the multistate epidemic of spinal and paraspinal *Exserohilum rostratum* infections following contaminated steroid injections [7]. In addition, an unusual case of intradural abscess due to *Candida dubliniensis* has been reported after epidural anesthesia, underscoring that invasive spinal procedures can serve as a route for fungal inoculation even in immunocompetent hosts [8].

*C. albicans*, the most frequently isolated fungal pathogen in vertebral osteomyelitis, is typically associated with immunocompromised states, chronic indwelling devices, or recent spinal instrumentation [6,9]. By contrast to rare fungal spinal infection following spinal injection, postoperative *Candida spondylodiscitis* has been more frequently documented, with a recent systematic review showing a two-year survival of ~80% and improved outcomes with prolonged antifungal therapy [2].

Unlike most reported cases, our patient developed a fungal infection despite being immunocompetent. Even in immunocompetent individuals, infection can develop for three reasons: hematogenous dissemination from distant sites, direct inoculation during procedures or trauma, and contamination through blood products [10]. In our case, direct inoculation is considered the most likely mechanism, as contaminated injectates, needles, or even inadequate hand hygiene by medical personnel can directly introduce fungi. Prevention of direct inoculation of fungi during spinal procedures requires strict aseptic technique, including proper hand hygiene, sterile barrier precautions, and effective skin disinfection with chlorhexidine-alcohol [11]. In addition, the exclusive use of sterile single-dose injectates and meticulous handling of needles and surgical instruments are essential, as highlighted by the 2012 multistate outbreak of *E. rostratum* linked to contaminated steroid vials [7].

Although antibiotics had been discontinued for three days before sampling, cultures of blood, urine, sputum, stool, and bone biopsy specimens failed to identify any organisms. Regarding the cause of this, we presume that it was attributable to (i) the patient’s prolonged use of intravenous broad-spectrum antibiotics for more than two months prior to biopsy, which likely suppressed bacterial or fungal growth, especially in well-vascularized tissue, and/or (ii) the localized nature of the infection in the intervertebral disc without systemic dissemination, which limited pathogen recovery from blood samples or bone biopsy. There is accumulating evidence that prolonged antibiotic therapy before specimen collection significantly lowers the likelihood of isolating causative organisms in osteomyelitis and spondylodiscitis [1,3]. Prolonged prior antibiotic use in our patient likely obscured early pathogen identification rather than serving as a direct risk factor for fungal infection. This observation is supported by previous reports, emphasizing that clinicians should maintain a high index of suspicion for fungal infections regardless of immune status [4]. Similarly, localized infections without hematogenous spread are well recognized to produce negative blood or urine cultures [4]. Furthermore, prolonged empirical antibiotic therapy has been implicated in the growing problem of antimicrobial resistance [5,12,13]. These findings collectively support the importance of early pathogen identification before initiating prolonged empirical therapy.

In our patient, multiple cultures, including blood, sputum, and bone biopsy with tissue Gram stain, AFB stain and culture, MTB PCR, PAS stain, and fungal culture, remained negative. Ancillary tests such as the serum β-D-glucan assay, which detects circulating fungal cell wall components in the blood, might have facilitated earlier identification, but they are not routinely performed in the initial work-up of post-procedural spinal infections due to limited specificity and cost considerations. β-D-glucan is a polysaccharide component of the fungal cell wall that is released into the bloodstream during invasive infection, and its detection serves as a non-culture-based biomarker. The absence of early β-D-glucan testing was a limitation in our case. When fungal infection is suspected, prompt use of this assay should be considered to facilitate the timely initiation of antifungal therapy [14].

Medical management, including culture-guided antimicrobial therapy, analgesia, and immobilization, is usually effective in infectious spondylitis [15,16]. In patients with progressive neurological deficits or failed empirical therapy, surgery may be necessary for decompression, stabilization, and pathogen identification [5,12,15]. In our case, however, the lack of early pathogen identification and targeted antifungal therapy allowed *C. albicans* to proliferate, causing neurological symptoms and necessitating surgery. We selected fluconazole because of its proven efficacy against *C. albicans*, favorable safety profile, and the absence of resistance in our institutional epidemiology. Amphotericin B or echinocandins were considered but were less suitable given the patient’s age and comorbidities. Treatment duration was planned for six weeks of intravenous therapy followed by oral continuation, consistent with published guidelines [17]. If the pathogen had been identified earlier, antifungal therapy might have obviated the need for surgical intervention. Surgery carries risks, including general anesthesia complications and potential long-term spinal instability, which can result in chronic pain [13,17]. This highlights the importance of early pathogen-directed therapy to optimize outcomes and minimize invasive procedures.

Interestingly, although the TFESI was performed at L5, the infection was localized at L1-L2. This discrepancy may be explained by the vertebral vascular anatomy. Vertebral bodies receive segmental arterial supply from the aorta, and valveless venous networks (Batson’s plexus) allow bidirectional flow, facilitating hematogenous spread of pathogens [12,13]. In transient candidemia, potentially related to mucosal barrier injury, prior malignancy, or prior antibiotic exposure, fungal seeding can occur at vertebral levels distant from the procedural site [4]. Injection-related contamination may further propagate infection via hematogenous routes or fascial planes. Recognition of these anatomical and pathophysiological considerations is essential, particularly when dealing with indolent pathogens such as *C. albicans*.

A key limitation of this study is that, as a single case report, a definitive causal relationship between the TFESI and the subsequent infection cannot be established, underscoring the necessity for further large-scale, controlled studies to better elucidate potential associations.

## Conclusions

This case emphasizes the critical importance of obtaining a definitive pathogen identification before initiating empirical antimicrobial therapy in patients with suspected spinal infection. Fungal pathogens such as *C. albicans *should be kept in mind as a possible etiology, even in patients without overt immunosuppression. Early biopsy and microbiological confirmation may play a role in guiding appropriate treatment, optimizing neurological outcomes, and avoiding unnecessary surgical intervention.
